# A Quality Initiative to Improve Appropriate Medication Dosing in Pediatric Patients with Obesity

**DOI:** 10.1097/pq9.0000000000000741

**Published:** 2024-06-11

**Authors:** Colleen P. Cloyd, Danielle Macedone, Jenna Merandi, Shawn Pierson, Maria Sellas Wcislo, Jeffrey Lutmer, Jennifer MacDonald, Onsy Ayad, Lindsay Kalata, R. Zachary Thompson

**Affiliations:** From the * Department of Pharmacy, Nationwide Children’s Hospital, Columbus Ohio; † Department of Critical Care Medicine, Nationwide Children’s Hospital, Columbus, Ohio; ‡ Nationwide Children’s Hospital Center for Clinical Excellence, Columbus, Ohio; § The Ohio State University College of Medicine, Columbus, Ohio.

## Abstract

**Introduction:**

Emerging evidence supports the use of alternative dosing weights for medications in patients with obesity. Pediatric obesity presents a particular challenge because most medications are dosed based on patient weight. Additionally, building system-wide pediatric obesity safeguards is difficult due to pediatric obesity definitions of body mass index-percentile-for-age via the Center for Disease Control growth charts. We describe a quality initiative to increase appropriate medication dosing in inpatients with obesity. The specific aim was to increase appropriate dosing for 7 high-risk medications in inpatients with obesity ≥2 years old from 37% to >74% and to sustain for 1 year.

**Methods:**

The Institute for Healthcare Improvement model for improvement was used to plan interventions and track outcomes progress. Interventions included a literature review to establish internal dosing guidance, electronic health record (EHR) functionality to identify pediatric patients with obesity, a default selection for medication weight with an opt-out, and obtaining patient heights in the emergency department.

**Results:**

Appropriate dosing weight use in medication ordered for patients with obesity increased from 37% to 83.4% and was sustained above the goal of 74% for 12 months.

**Conclusions:**

Implementation of EHR-based clinical decision support has increased appropriate evidence-based dosing of medications in pediatric and adult inpatients with obesity. Future studies should investigate the clinical and safety implications of using alternative dosing weights in pediatric patients.

## INTRODUCTION

Pediatric obesity is a growing epidemic in the United States. The Centers for Disease Control (CDC) estimates that 19.7% of children and adolescents between the ages of 2 and 20 years meet obesity criteria.^1^ The American Academy of Pediatrics recently published clinical practice guidelines for evaluating and treating this vulnerable patient population.^2^ Dosing medications in this population presents a unique problem. Most pediatric medications are dosed using the patient’s weight per kilogram (eg, mg/kg) or medication dose per meters squared (eg, mg/m^2^). Furthermore, due to differences in body composition, metabolism, and elimination in a patient with obesity, certain medications may be distributed and processed differently. These factors put children with obesity at an increased risk of drug toxicity when using weight-based dosing.^3–5^

There is growing evidence for appropriate dosing of certain medications in children with obesity.^3,6–9^ Two alternative dosing weights are used for dosing medications in this population: an ideal body weight, which is a calculation of the lean body mass for a patient’s age and height, and an adjusted body weight, which is a calculation that accounts for some proportion of excess body mass.^6,10^ The most appropriate weight for a specific medication is determined by the drug’s pharmacokinetic and pharmacodynamic properties.

Although evidence exists to support the use of alternative dosing weights in pediatric patients with obesity, the logistics of embedding related clinical decision support into the electronic health record (EHR) remains challenging. It has not been described in the literature. One barrier is that obesity in children is defined differently than in adults. In adults, a BMI of ≥30 meets the criteria for obesity, whereas pediatric obesity is determined using the child’s BMI-percentile-for-age. This BMI-percentile-for-age is calculated using the patient’s weight, height, and age-specific growth chart and compared with other children of the same sex and age. Children with a BMI-percentile-for-age of greater than the 95^th^ percentile meet the criteria for obesity and may require dosing adjustments of some medications.^3,6,7,11^ Incorporating this specificity into EHR-based clinical decision support is challenging to standardize across all patients and relevant medications.

The global goal of this quality improvement initiative was to improve medication dosing in patients with obesity via behavioral economic concepts of defaults and nudges leveraged within the EHR. The specific aim of this quality initiative was to increase the appropriate dose of seven intravenous medications in pediatric and adult inpatients with obesity over 2 years old from 37% to >74% and sustain this increase for one year.

## METHODS

### Context

Our hospital is a 673-bed, free-standing pediatric tertiary care hospital in the Midwestern United States, and we use an EHR for patient care (Epic Systems Corporation, Verona, Wisconsin). Before implementing this initiative, we did not have formal recommendations for using alternative dosing weights in patients with obesity.

### Pre-intervention

The first step of this initiative was the development of an internal document summarizing current literature (up to date as of January 2021) on dosing of 31 medications in patients with obesity (**See table, Supplemental Digital Content 1**, which shows alternative dosing weight recommendations. http://links.lww.com/PQ9/A568).^3–9,11–34^ Medications were included in this document if they had high potential for serious patient harm if inappropriately dosed on an mg/kg basis in a patient with obesity and if they had frequent utilization at our institution. A pharmacist-led multidisciplinary task force reviewed the literature and decided which alternative weight (ideal body weight or adjusted body weight) would be most appropriate for each medication when dosed in patients with obesity. This document was reviewed and approved by physician, pharmacist, and nursing provider champions from various inpatient hospital specialties before implementation.

**Fig. 1.**
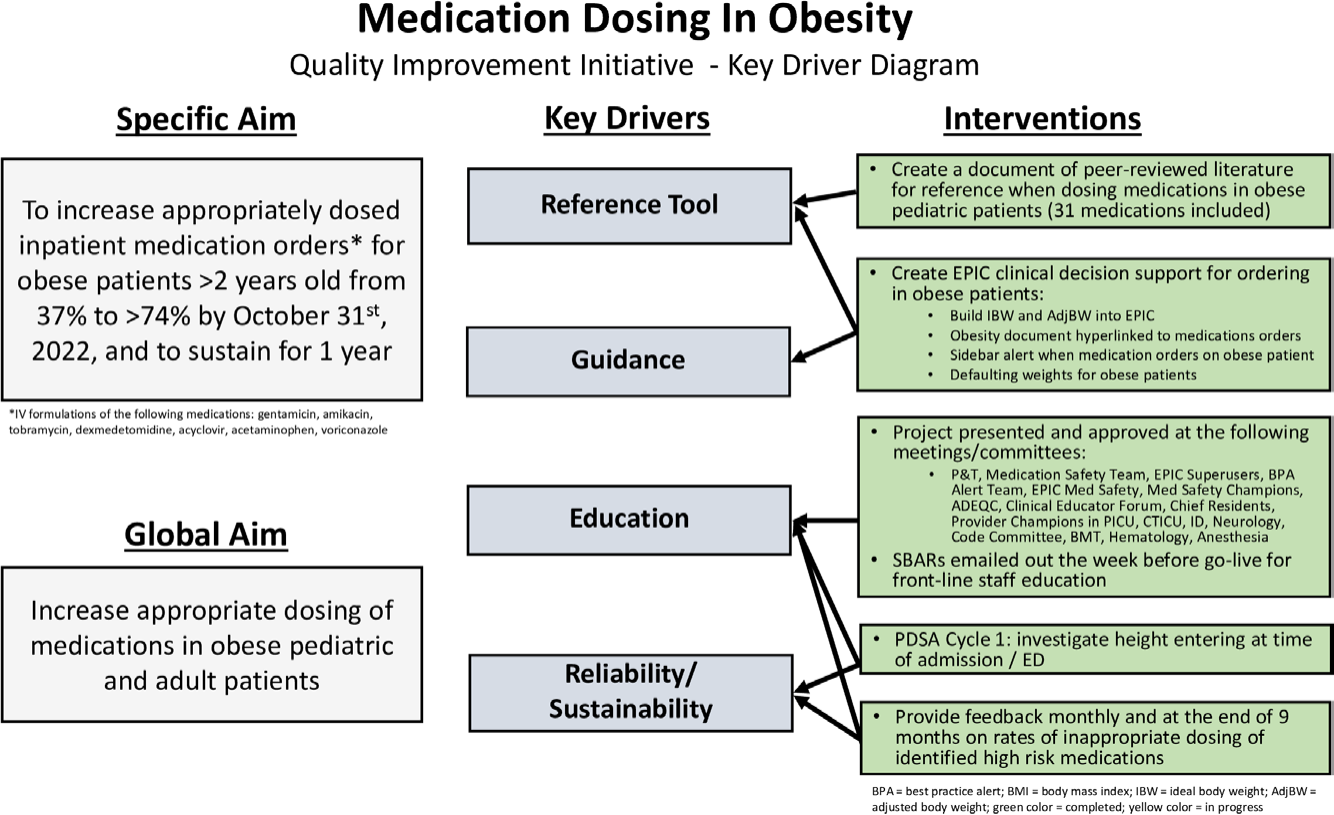
Key driver diagram. ADEQC, adverse drug event quality collaborative; CTICU, cardiothoracic intensive care unit; PICU, pediatric intenive care unit; ID, infectious disease; BMT, bone marrow transplant; SBARs, situation, background, assessment, recommendation; PDSA, plan, do, study, act.

A multidisciplinary team, including pharmacists, critical care physicians, a medication safety pharmacist, information services, a data analyst, and a QI specialist, used strategies from the Institute for Healthcare Improvement model for improvement to establish an aim statement, key driver diagram, and plan-do-study-act cycles (Fig. 1).

### Interventions

To identify patients with obesity greater than 2 years old, the EHR was programmed to include criteria for obesity using CDC definitions: greater than the 95^th^ percentile BMI-for-age for pediatric patients <20 years old and an absolute BMI of >30 for those ≥20 years old. The Traub equation^10^ was used to calculate pediatric ideal body weight in patients less than 60 inches (152.4 cm) tall, allowing EHR calculation of ideal and adjusted body weight for all patients ≥2 years old. We used a cofactor of 0.4 for all adjusted body weight calculations.

**Fig. 2.**
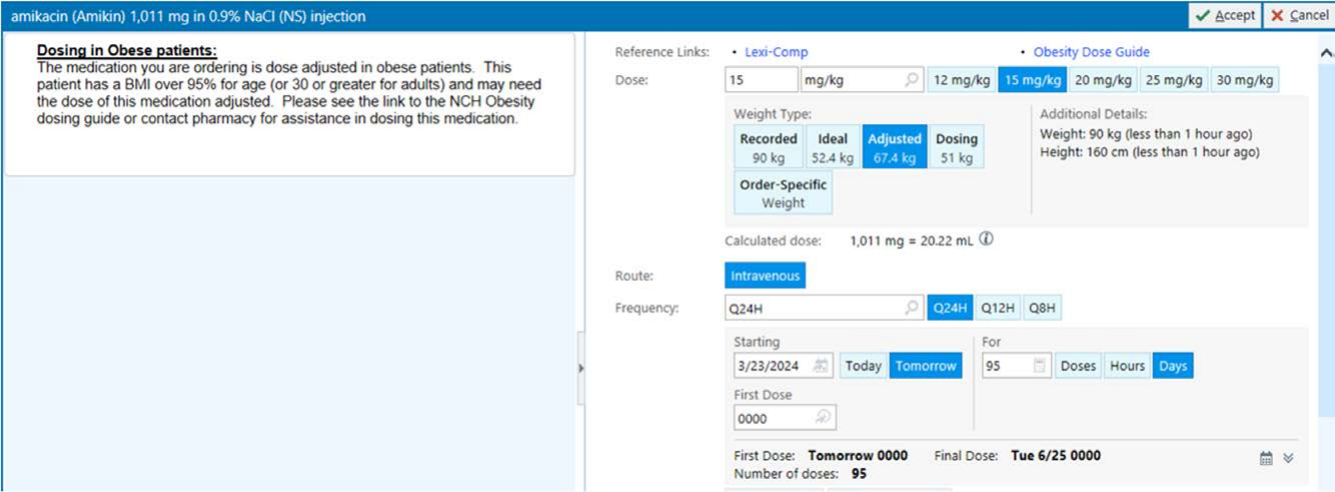
EHR functionality: example medication order for a patient with obesity. Functionality of the primary intervention included a passive alert alongside the medication order stating the patient has obesity, a hyperlink to our institution’s obesity dosing guideline, and an opt-out functionality that defaults the dosing weight used to the preferred alternative dosing weight.

Upon ordering, the EHR provided clinical decision support if obesity criteria were met, for all 31 medications on our internal obesity dosing guideline. The ordering provider was notified that the patient met obesity criteria and an alternative dosing weight should be considered. This information was presented passively alongside the medication order. When possible, the EHR automatically preselected the appropriate alternative weight, leveraging an “opt-out” strategy for weight selection. A hyperlink to the obesity medication dosing guideline was also embedded in the order. Figure 2 is an example of a medication ordered for an obese patient and highlights the functionalities described.

For seven selected high-risk intravenous medications (amikacin, gentamicin, tobramycin, acetaminophen, dexmedetomidine, voriconazole, and acyclovir), additional support related to medication dosing for obesity was also added to the pharmacy order verification process. For these seven medications, an alert requiring a manual override was triggered when a pharmacist verified an order for a patient with obesity. These medications were chosen for this additional alert due to the strength of evidence for alternative dosing in patients with obesity and they have been identified as offending agents in previous medication safety events for patients with obesity at our institution. By limiting this additional alert to these medications, we sought to minimize alert fatigue in ordering providers and pharmacists. All EHR functionalities described were custom-built using standard Epic modules and without specific vendor assistance.

### Study of the Interventions and Measures

#### Retrospective Determination of Appropriate Medication Dosing

All inpatient orders for the medications of interest in patients with obesity greater than 2 years of age were collected for the 12 months leading up to implementation of the EHR change interventions. To obtain baseline data, the obesity dosing rules were applied retrospectively to assess appropriate medication dosing. If the patient had obesity, we determined if the appropriate alternative dosing weight was used on the medication order. Orders were excluded from analysis if: (1) Flat (mg) doses of aminoglycoside antibiotics (amikacin, gentamicin, and tobramycin) and voriconazole were based on previous drug levels in that patient or (2) the patient’s height and weight were plotted on a growth chart other than the CDC 2-20-year-old chart. The appropriateness of flat doses (mg) of acetaminophen, acyclovir, voriconazole, amikacin, gentamicin, and tobramycin was determined by dividing the dose in milligrams by the patient’s adjusted or ideal body weight. If this calculated milligram per kilogram dose was within the LexiComp^30^ guidelines for dosing that medication, it was considered appropriate.

#### Outcome Measure

The primary outcome measure was the percentage of medication orders using the recommended (ie, appropriate) dosing weight for seven chosen medications: amikacin, gentamicin, tobramycin, acetaminophen, dexmedetomidine, voriconazole, and acyclovir (Table 1). These medications were selected as described in the methods section. The goal of >74% appropriate dosing was chosen as it would represent a doubling of proper dosing of baseline. It was also higher than any historical monthly performance in our baseline data.

**Table 1.** Alternative Dosing Weight

Medication	Dosing Weight Recommendation	Max Dose/Max Starting Dose
Acetaminophen	Adjusted body weight	1000 mg
Acyclovir	Ideal body weight	800 mg
Amikacin	Adjusted body weight	2000 mg, consider 1^st^ dose levels
Dexmedetomidine	Adjusted body weight	NA
Gentamicin	Adjusted body weight	700 mg, consider 1^st^ dose levels
Tobramycin	Adjusted body weight	720 mg, consider 1^st^ dose levels
Voriconazole	Ideal body weight	400 mg

#### Process Measures

Appropriate medication dosing was analyzed using various factors, including patient age, specific medication, and hospital unit. Pareto charts were created for each factor to identify opportunities for additional interventions following the initial EHR change. Additionally, a funnel chart was used to evaluate inappropriate dosing by unit. This analysis highlighted additional opportunities within the emergency department (ED) and the cardiothoracic intensive care unit. Further investigation found that obtaining patient heights in the ED was a barrier to determining obesity. Targeted interventions were pursued for this unit specifically. We provided education on the importance of height measurement for the calculation of pediatric obesity. We ordered new stadiometers for all ED intake rooms, which could simultaneously record both the weight and height for all ambulatory children, and we added height as an entry on all electronic ED intake forms.

#### Analysis

An SPC control chart (p chart) was used to monitor the outcome measure over time following interventions implementation. The chart was updated monthly and analyzed for signals of special cause variation using established American Society for Quality criteria. The data were reviewed during quarterly multidisciplinary QI team meetings.

#### Safety Considerations/Balancing Measure

Individual medication safety events submitted through our hospital voluntary reporting system related to obesity dosing were reviewed by the QI team throughout the project to ensure we were not instilling significant safety events into our system.

#### Ethical Considerations

This initiative was deemed quality improvement, which per institutional policy, was exempt from IRB review. This project was reviewed and approved by the hospital Pharmacy and Therapeutics and Medication Safety Committees, and education was provided to all key stakeholders (including physicians, nurse practitioners, nurses, and pharmacists) throughout the hospital before implementation. This project focused on creating medication dosing guidance and associated EHR functionality, and all applicable medication orders in patients with obesity were equally subject to the effects of interventions. The authors followed the Standards for Quality Improvement Reporting Excellence Guidelines for this publication.^35^

## RESULTS

A total of 3,105 medication orders were assessed for appropriate weight in 1,201 unique patients with obesity. Group demographics, before and after implementation of EHR changes, and the number of orders for each drug evaluated are found in Tables 2 and 3. A student *t* test for age and absolute BMI between pre and postintervention groups was run, and a *P* value of <0.05 demonstrated the postimplementation group was older. Acetaminophen comprised more than 50% of the medication orders assessed.

**Table 2.** Demographics

	Pre-implementation (n = 375 Patients)	Postimplementation (n = 826 Patients)	*P*
% Male	54.6	50.1	
Age in years, median, (IQR)	11.5 (6.0–15.3)	12.6 (7.53–16.3)	0.003
Absolute BMI, median, (IQR)	28.1 (21.5–33.6)	28.7 (23.7–34.8)	0.34

**Table 3.** Medication Orders

	Pre-implementation (n = 816 Orders)	Postimplementation (n = 2289 Orders)
**Medication Orders**, n (%*)		
Acetaminophen	514 (62.9)	1330 (58.1)
Dexmedetomidine	191 (23.4)	658 (28.8)
Amikacin	43 (5.3)	130 (5.7)
Acyclovir	32 (3.9)	58 (2.5)
Gentamicin	21 (2.6)	89 (3.9)
Voriconazole	9 (1.1)	24 (1.0)
Tobramycin	6 (0.7)	0 (0)

* Percentages may not add up to 100 due to rounding.

**Fig. 3.**
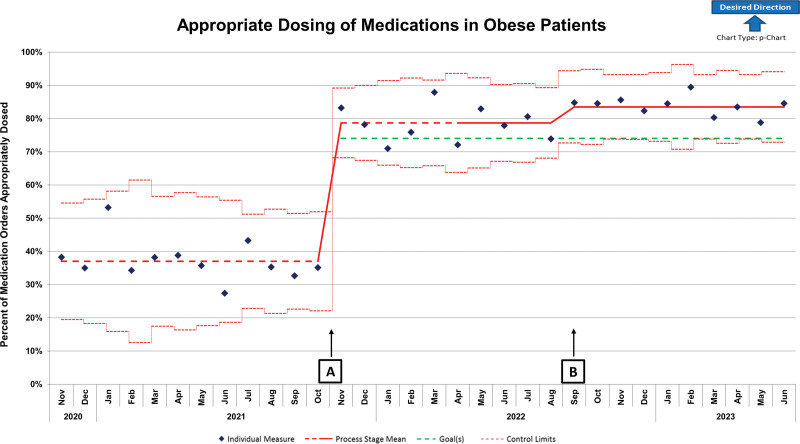
P chart for percentage of appropriate dosing of select medications in patients with obesity. Hospital-wide appropriate dosing of medications (amikacin, gentamicin, tobramycin, acetaminophen, dexmedetomidine, voriconazole, and acyclovir) in obese patients before and after interventions. Intervention A: Implementation of EHR functionality, obesity dosing guideline, and hospital-wide education. Intervention B: Implementation of obtaining heights in the ED, new stadiometers in all ED intake rooms, staff education, and adding a height entry to all electronic ED intake forms.

**Fig. 4.**
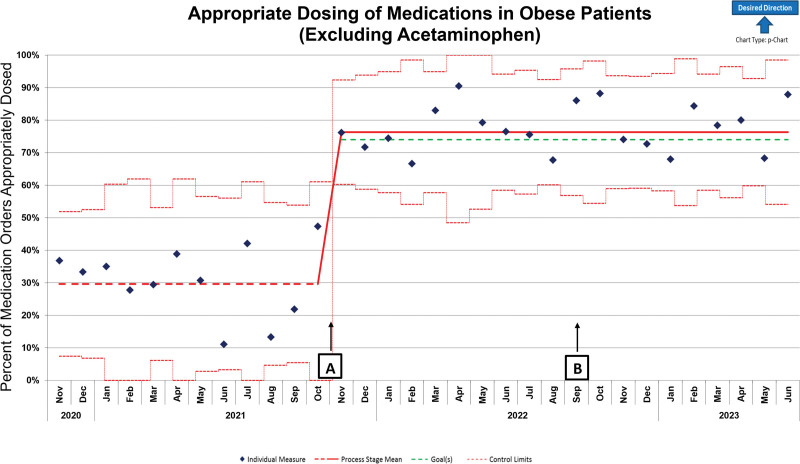
P chart for percentage of appropriate dosing of select medications in patients with obesity excluding acetaminophen orders. Hospital-wide appropriate dosing of medications (excluding acetaminophen) in obese patients before and after interventions. Intervention A: Implementation of EHR functionality, obesity dosing guideline, and hospital-wide education. Intervention B: Implementation of obtaining heights in the ED, new stadiometers in all ED intake rooms, staff education, and adding a height entry to all electronic ED intake forms.

Before implementation of EHR changes, 37% of medication orders were appropriately dosed in patients with obesity ≥2 years of age. Upon implementation of EHR clinical decision support, the appropriate use of an alternative dosing weight increased above the goal of 74%, with an average of 78.7% orders appropriately dosed. Implementation of the EHR changes resulted in a centerline shift based on the SPC criteria of 8 consecutive points above the centerline (Fig. 3). A secondary control chart excluded acetaminophen orders due to this medication comprising >50% of orders assessed (Fig. 4). Without acetaminophen orders, before EHR changes 29.7% of orders were appropriately dosed, and after EHR changes 77% of orders were appropriately dosed.

**Fig. 5.**
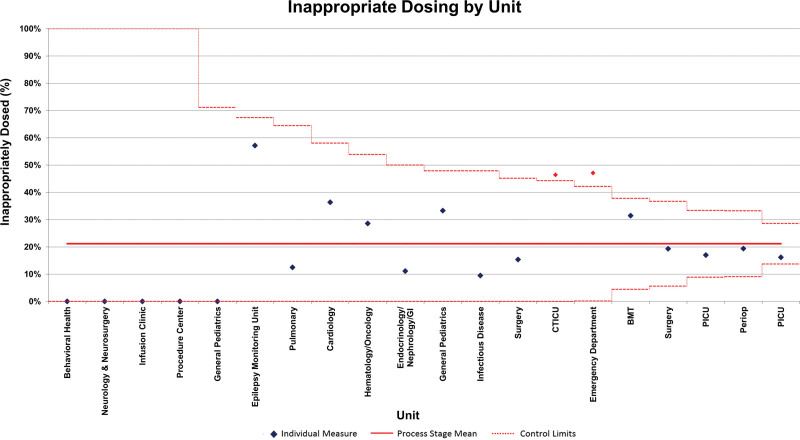
Funnel chart of inappropriate dosing by hospital unit/service.

Process measure results demonstrated that the ED had significantly more inappropriate dosing than expected compared with other hospital units (Fig. 5). Following implementation of targeted interventions to obtain height measurements in the ED for all children ≥2 years of age, appropriate dosing specifically for the ED increased from 48% to 92%. Hospital wide, these changes resulted in another centerline shift based on the SPC criteria of 8 consecutive points above the centerline to an average of 83.4% of medication orders appropriately dosed (Fig. 3). The changes in appropriate dosing of medications have been sustained for 20 months.

Before implementation, one safety event was reported related to obesity dosing for one of our studied medications among 2613 total medication safety reports submitted (rate of 0.000383). Post-implementation through the end of the study period, 6 events were reported out of a total of 4611 (rate of 0.001301).

## DISCUSSION

### Interpretation

We are the first children’s hospital to report the use of the EHR to identify and guide providers in medication dosing for pediatric patients with obesity. This quality initiative uses concepts of behavioral economics^36^ via leveraging the EHR to drive correct medication dosing in patients with obesity. Multiple behavioral biases might cause a provider to not choose the correct dosing weight for a medication. There is a cognitive strain needed to remember which medications need to be dosed differently in patients with obesity and which alternative dosing weight should be used. We provide this information to our providers at the time of ordering. Also, decision and alert fatigue would mean an extra alert to drive practice would likely result in minimal change, so we sought to minimize additional alerts to the provider with this intervention. Finally, by leveraging opt-out functionality, we nudge the behavior and tendency towards prioritizing efficiency in work by using shortcuts, allowing these decisions to be more streamlined for our front-line providers.^37^ By providing an opt-out weight selection for patients with obesity, we are nudging our providers towards best practice while still allowing the autonomy to choose otherwise, if that is determined as best for the patient. This use of default settings is highly effective in changing physician prescribing behavior, and we have demonstrated this again with this quality initiative.^38,39^ Behavioral economics concepts are becoming more frequently used in healthcare decision-making, and this project highlights their use to effectively drive prescribing practice in a vulnerable patient population.

Guided by information from our department-specific assessment of appropriate dosing as described above, ten months after implementing these EHR changes, height measurement became much more common for ED patients. With this change in September of 2022, we saw an increase in our appropriate dosing of medications overall, specifically, within the ED. This is likely due to the ED being the source for most admissions to the inpatient hospital, and many medications ordered in the ED are continued upon admission. Furthermore, obtaining a height in the ED allows the EHR to identify obese patients early in their encounter and subsequently provide dosing nudges and defaults for medication dosing as appropriate.

### Limitations

There are several limitations to address. First, the literature on this subject is still sparse. Clinical outcome studies in patients with obesity dosed on alternative dosing weights versus actual body weight should be conducted to fully elucidate this intervention’s clinical implications.

We limited the assessment of EHR changes to seven medications instead of all 31 medications on the obesity dosing list. This decision was made to support the feasibility of this initiative from a data collection and analysis standpoint. There is a chance that the remaining 24 medications may have had a different outcome than we observed with these seven medications. In addition, the number of orders for acetaminophen in patients with obesity was much higher than the other six medications assessed. To assess this, we ran a second control chart, removing all acetaminophen orders from the analysis. Still, we observed a centerline shift in appropriate dosing with the implementation of EHR functionality. Of note, in this secondary analysis we did not observe a second centerline shift following the interventions made in the ED. This is because the number of orders assessed monthly is too small to identify the minor shift in appropriate dosing after the ED interventions. Importantly, the maximum dose of acetaminophen at our institution is 1,000 mg per dose. By that definition, if the patient’s adjusted body weight is >66 kg, this is counted as an appropriate flat dose to administer to the patient. This would lead to higher appropriate doses in our baseline data but would have the same effect on postimplementation data, so it does not contribute to the overall change in the appropriateness of dosing. As noted, our groups were statistically different in age with the postimplementation group being older.

We did not perform any balancing measures for safety analysis during this initiative but followed medication safety events throughout the intervention and performed an ad hoc analysis at completion. We saw a slight increase in the absolute number of medication events related to obesity dosing, which can be due to increased awareness of this initiative and change in medication dosing practice. The rate of safety events of less than 0.15% related to obesity dosing remained very low after the intervention. The formal measurement of medication safety event rates using QI methodology during the active project should be considered by others when initiating similar projects.

Lastly, medication orders were excluded from analysis if the patient’s height and weight were graphed using an alternative growth chart. A subset of these patients likely still has obesity with altered body composition and drug distribution; it will be important to analyze this group in more depth in the future.

## CONCLUSIONS

Using a novel EHR build, we have increased appropriate dosing of select medications with published evidence supporting alternative weight dosing for pediatric and adult inpatients with obesity from 37% to 83.4% and have sustained this level for more than 1 year. This quality initiative shows that by leveraging technology with both passive and limited interruptive clinical decision support it is possible to elicit immediate and sustained improvement.

## Supplementary Material


