# Supplementary material for: A Quality Initiative to Improve Appropriate Medication Dosing in Pediatric Patients with Obesity
Source: Pediatr Qual Saf. 2024 Jun 11;9(3):e741. doi: 10.1097/pq9.0000000000000741 (PMC11167219; doi:10.1097/pq9.0000000000000741)
Supplement: Supplementary file 1 [file pqs-9-e741-s001.pdf]

Supplemental Table 1: Alternative Dosing Weight

Medication	Dosing Weight Recommendation
Acetaminophen*	AdjBW
Acyclovir*	IBW
Albumin 25%	IBW
Amikacin*	AdjBW
Aminophylline (Theophylline)	Loading dose: ActualBW<br>Maintenance: IBW
Baclofen	IBW
Calcium Chloride	IBW, when dosed for electrolyte replacement
Calcium Gluconate	IBW, when dosed for electrolyte replacement
Cisatracurium	IBW
Dexmedetomidine*	AdjBW
Enoxaparin	ActualBW, consider AdjBW if BMI >40
Fentanyl	AdjBW
Gentamicin*	AdjBW
Heparin	AdjBW
Hydromorphone	AdjBW
Ibuprofen	AdjBW
Immune Globulin (IVIG)	IBW
Ketamine	IBW
Mannitol	IBW
Methylprednisolone	IBW
Midazolam	IBW, may consider ActualBW for bolus doses if intubated
Morphine	IBW
Phenytoin / Fosphenytoin	Loading dose: ActualBW<br>Maintenance: AdjBW
Propofol	Loading/induction dose: AdjBW<br>Maintenance: ActualBW
Phenobarbital	ActualBW
Rocuronium	IBW
Sodium Bicarbonate	IBW, when used for pH/electrolyte replacement
Tobramycin*	AdjBW
Vecuronium	IBW
Vancomycin	ActualBW
Voriconazole*	IBW

*medication assessed in this quality initiative; ActualBW = actual body weight; AdjBW = adjusted body weight; IBW = ideal body weight; AdjBW calculations all use a cofactor=0.4
